# α-Conotoxins Enhance both the In Vivo Suppression of Ehrlich carcinoma Growth and In Vitro Reduction in Cell Viability Elicited by Cyclooxygenase and Lipoxygenase Inhibitors

**DOI:** 10.3390/md18040193

**Published:** 2020-04-07

**Authors:** Alexey V. Osipov, Tatiana I. Terpinskaya, Tatsiana Yanchanka, Tatjana Balashevich, Maxim N. Zhmak, Victor I. Tsetlin, Yuri N. Utkin

**Affiliations:** 1 Shemyakin-Ovchinnikov Institute of Bioorganic Chemistry, Russian Academy of Sciences, ul. Miklukho-Maklaya 16/10, 117997 Moscow, Russia; osipov-av@ya.ru (A.V.O.); mzhmak@gmail.com (M.N.Z.); victortsetlin3f@gmail.com (V.I.T.); 2 Institute of Physiology, National Academy of Sciences of Belarus, ul. Akademicheskaya, 28, 220072 Minsk, Belarustanyaya190@gmail.com (T.Y.); tbalashevich@bk.ru (T.B.)

**Keywords:** cyclooxygenase, Ehrlich carcinoma, inhibitors, lipoxygenase, α-conotoxin

## Abstract

Several biochemical mechanisms, including the arachidonic acid cascade and activation of nicotinic acetylcholine receptors (nAChRs), are involved in increased tumor survival. Combined application of inhibitors acting on these two pathways may result in a more pronounced antitumor effect. Here, we show that baicalein (selective 12-lipoxygenase inhibitor), nordihydroguaiaretic acid (non-selective lipoxygenase inhibitor), and indomethacin (non-selective cyclooxygenase inhibitor) are cytotoxic to Ehrlich carcinoma cells in vitro. Marine snail α-conotoxins PnIA, RgIA and ArIB11L16D, blockers of α3β2/α6β2, α9α10 and α7 nAChR subtypes, respectively, as well as α-cobratoxin, a blocker of α7 and muscle subtype nAChRs, exhibit low cytotoxicity, but enhance the antitumor effect of baicalein 1.4-fold after 24 h and that of nordihydroguaiaretic acid 1.8–3.9-fold after 48 h of cell cultivation. α-Conotoxin MII, a blocker of α6-containing and α3β2 nAChR subtypes, increases the cytotoxic effect of indomethacin 1.9-fold after 48 h of cultivation. In vivo, baicalein, α-conotoxins MII and PnIA inhibit Ehrlich carcinoma growth and increase mouse survival; these effects are greatly enhanced by the combined application of α-conotoxin MII with indomethacin or conotoxin PnIA with baicalein. Thus, we show, for the first time, antitumor synergism of α-conotoxins and arachidonic acid cascade inhibitors.

## 1. Introduction

Tumor cells are characterized by the increased activity of some biochemical pathways that promote their survival and proliferation. One of these pathways is the arachidonic acid cascade: arachidonate is released from the phospholipids of the cell membrane and then transformed into eicosanoids. Among the latter are prostanoids (prostaglandins and thromboxanes) formed with the participation of cyclooxygenases (COX), and leukotrienes formed with the participation of lipoxygenases (LOX). The tumor-promoting effect of COX is associated with the increased PGE2 synthesis, which has an immunosuppressive effect [1] and plays an important role in the growth and progression of tumors. A number of compounds, formation of which is catalyzed by LOX, contribute to the growth of tumors as well. Thus, leukotriene B4 and hydroxyeicosotetraenoic acids (HETE), such as 5S-HETE and 12S-HETE, stimulate the proliferation and survival of tumor cells, metastasis, neoangiogenesis, and immunosuppression of the tumor microenvironment [2,3]. Noteworthily, COX and LOX inhibitors exhibited antitumor effects in experimental animal and clinical studies [4,5].

One of the features pertinent to many types of tumors is the increased expression of nicotine acetylcholine receptors (nAChRs). A combination of different nAChR subunits results in the formation of multiple subtypes of this receptor, which can be heteromeric, comprising different subunits, or homomeric, consisting of identical subunits. Along with the cells of neuronal origin, nAChRs are expressed in several types of non-neuronal cells and participate in the regulation of many cellular functions. In particular, these receptors are involved in enhancing the proliferation and survival of tumor cells and in accelerating tumor growth [6]. In this regard, nAChR blockers may be considered as antitumor agents.

α-Conotoxins from the venom of sea snails of the Conus genus are selective blockers of these particular nAChR subtypes and therefore can be expected to manifest antitumor activity. Indeed, we have previously shown that some α-conotoxins can inhibit the development of Ehrlich carcinoma, the most active being α-conotoxin MII [7].

One of the approaches to the treatment of malignant tumors is the use of drugs that block their survival pathways. However, the inhibition of one of the survival mechanisms in cancer cells is often compensated by other ones, which reduce the antitumor effect. Therefore, the use of several drugs acting on different molecular targets (for example, both on the arachidonic acid cascade and the nAChRs) may result in a more pronounced effect.

In this paper, we describe the effect of several α-conotoxins on the ability of COX and LOX inhibitors to reduce the viability of Ehrlich ascite carcinoma (EAC) cells in vitro and suppress the growth of this tumor in vivo. In particular, α-conotoxin MII (specific inhibitor of α6 and α3β2 nAChR subtypes), PnIA (specific to α3β2 and α6β2 subtypes), RgIA (to α9α10) and ArIB11L16D (to α7) were used in this study. In addition, we have utilized α-cobratoxin from cobra venom, a blocker of α7 and muscle type nAChRs, with the information about the antitumor effects of this toxin being controversial [8]. As COX and LOX inhibitors, we used indomethacin, a non-selective COX inhibitor, SC-560, a selective COX-1 inhibitor, NS-398, a selective COX-2 inhibitor, nordihydroguaiaretic acid (NDGA), a non-selective LOX inhibitor, zileuton, a 5-LOX inhibitor, and baicalein, a 12-LOX inhibitor. It was found that α-conotoxins enhance the anti-tumor activity of COX and LOX inhibitors.

## 2. Results

### 2.1. Influence of α-Conotoxins and α-Cobratoxin in Combination with COX and LOX Inhibitors on Viability of EAC Cells

To assess the influence of α-conotoxins on the effects of COX and LOX inhibitors on the viability of EAC cells, the substances from each group (α-conotoxins and inhibitors) were incubated with cells first individually and then in binary mixtures containing one substance from each group. It was found that after 24–48 h of cell incubation with nAChR blockers, they either did not affect the viability of EAC cells or decreased it by 1% to 4% (Figure S1). Overall, these results are consistent with our previous data [9].

**Figure 1:**
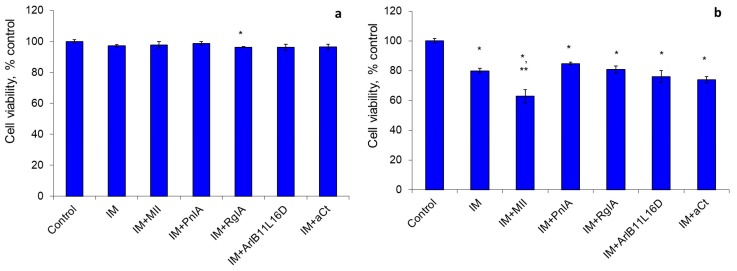
The effect of indomethacin (IM) and nicotinic acetylcholine receptor (nAChR) blockers on the viability of Erlich ascite carcinoma (EAC) cells after cultivation for 24 h (**a**) and 48 h (**b**). * *p* < 0.05 compared to control; ** *p* < 0.05 compared to the series “IM”.

The indomethacin, a non-selective COX inhibitor, did not affect the cell viability after incubation for 24 h, and after 48 h it decreased the viability by 20%. α-Conotoxin MII enhanced this indomethacin effect by increasing the number of dead cells 1.9-fold (Figure 1).

**Figure 2:**
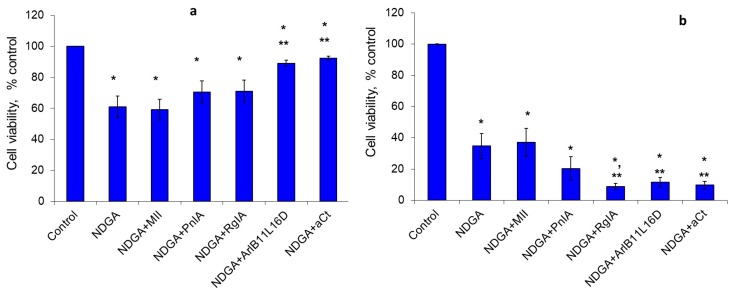
The effect of NDGA and nAChR blockers on the viability of EAC cells after cultivation for 24 h (**a**) and 48 h (**b**). * *p* < 0.05 compared to control; ** *p* < 0.05 compared to the series “NDGA”.

We found a high sensitivity in EAC cells to NDGA, a non-specific inhibitor of the LOX pathway, and also to baicalein, a 12-LOX inhibitor. After 24 h of cultivation, NDGA reduced the viability of tumor cells by almost 40%, and, after 48 h, 65% of the cells were dead. With the exception of conotoxin MII, all other nAChR blockers decreased the NDGA effect by 10%–31% after 24 h (Figure 2a) and increased it 1.8–3.9-fold after 48 h (Figure 2b).

**Figure 3:**
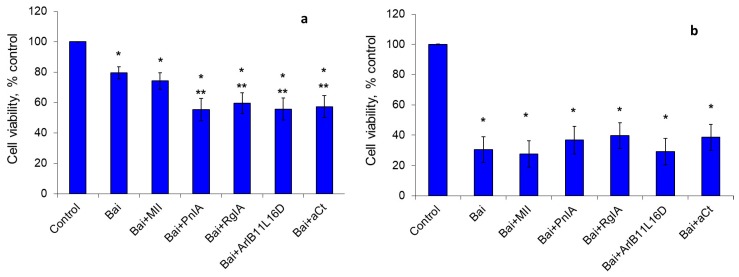
The effect of baicalein (Bai) and nAChR blockers on the viability of EAC cells after cultivation for 24 h (**a**) and 48 h (**b**). * *p* < 0.05 compared to control; ** *p* < 0.05 compared to the series “Bai”.

Baicalein, the 12-LOX inhibitor, reduced cell viability by 20% after cultivation for 24 h, and three-fold after 48 h. nAChR blockers enhanced the baicalein effect after 24 h of cultivation, reducing the percentage of viable cells 1.4-fold (Figure 3a). After 48 h of cultivation, no statistically significant influence of nAChR blockers on the baicalein effect was observed (Figure 3b).

NS-398, the selective COX-2 inhibitor, reduced the viability of tumor cells by 3% and 4% after 24 and 48 h of incubation, respectively, and nAChR blockers had virtually no impact on this NS-398 effect (Figure S2). SC-560, a selective COX-1 inhibitor, and zileuton, the 5-LOX inhibitor, either slightly decreased the viability of EAC cells (by 1%–3%) or produced no effects. No enhancement of negligible inhibitory effects of these inhibitors in the presence of nAChR blockers was observed (Figures S3 and S4).

### 2.2. Influence of α-conotoxins in Combination with COX and LOX Inhibitors on the EAC Growth

To examine the influence of the individual compounds under study or their combinations on EAC growth, mice were inoculated subcutaneously with EAC viable cells. Starting from the day of inoculation, animals were injected intraperitoneally for 4 weeks with an interval of 3–4 days; after that time, no injections were done.

For the experiments in vivo, we used those combinations of nAChR blockers with COX or LOX inhibitors that showed the pronounced cytotoxic effect in vitro. From among nAChR blockers, α-conotoxins MII, inhibiting α6 and α3β2 nAChR subtypes, and PnIA, blocker of α3β2 and α6β2 nAChR subtypes, were chosen for these experiments. The combinations of these α-conotoxins with indomethacin or NDGA, as well as of α-conotoxin PnIA with baicalein, were studied in detail. In parallel, the effect of each of these compounds alone was also examined.

**Figure 4:**
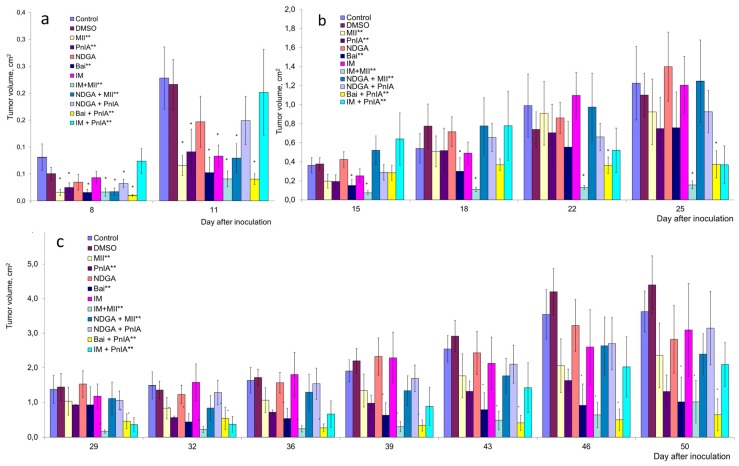
The effects of α-conotoxins, cyclooxygenase (COX) and lipoxygenase (LOX) inhibitors and their combinations on the growth of Ehrlich carcinoma in mice. The volume of tumor on days 8–11 (**a**), 16–25 (**b**) and 29–50 (**c**) after inoculation was determined. The effect of dimethyl sulfoxide (DMSO) which was used as a solvent for COX and LOX inhibitors was also presented. * *p* < 0.05 compared to DMSO at each individual stage of tumor growth (Mann–Whitney test); ** *p* < 0.05 using the ANOVA for the analysis of the entire series for 50 days of experiment.

Up to day 11 after tumor inoculation, application of all the studied drugs and their combinations resulted in a decrease in, or a tendency to decrease, tumor volume compared to the controls—blank or solvent injections (Figure 4a). On the following days, animals treated with indomethacin, NDGA or NDGA in combination with PnIA showed an acceleration in tumor growth, and the tendency to suppress tumor growth was not observed anymore (Figure 4b,c).

In other experimental series, the different degrees of tumor growth retardation were seen. The combination of NDGA with MII had some inhibitory effect on the tumor growth. Up to day 11 of the experiment, there was a noticeable decrease in the volume of the tumor (Figure 4a); however, the effect became weak in the following days. Nevertheless, it was statistically significant throughout the duration of the experiment.

When α-conotoxin MII or indomethacin were administered separately, tumor growth suppression was observed, but it was clearly visible only in the first two weeks after inoculation of tumor cells, resulting in a decrease in tumor volume 1.4–4-fold (Figure 4a). After that time, the effect of MII was weak, and the effect of indomethacin was completely abolished (Figure 4b,c). However, the most significant growth inhibition was produced by the combination of these two substances. In this series, at days 8–25 after inoculation, the average value of tumor volume decreased 3–8-fold compared to the controls (Figure 4a,b).

During the first 2 weeks post-inoculation, in tumor-bearing animals treated with α-conotoxin PnIA or baicalein alone, a 1.6–4-fold decrease in the average tumor volume was observed; afterwards, the trend remained, but was less pronounced. PnIA did not significantly alter the effect of baicalein in the early stages of tumor development (Figure 4a), but this combination was the most effective in the later stages of the experiment (Figure 4c, yellow columns). At the end of the experiment, this combination reduced the tumor volume six-fold.

**Figure 5:**
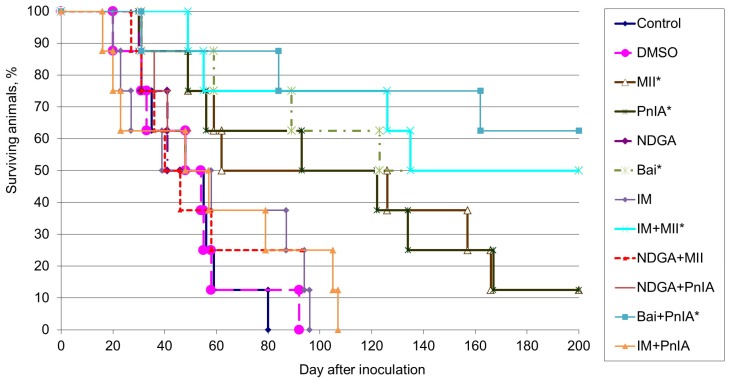
The effects of α-conotoxins MII and PnIA (0.1 nmole/kg), indomethacin (IM, 10 mmoles/kg), nordihydroguaiaretic acid (NDGA, 30 mmoles/kg), baicalein (Bai, 50 mmoles/kg) and their combinations on the survival of animals - carriers of Ehrlich carcinoma. Data for DMSO (1 ml/kg), which was used as a solvent, are shown as well. * *p* < 0.05 when compared with control (according to the Cox’s F-test).

We analyzed the influence of α-conotoxins and COX and LOX inhibitors on lifespan and survival rate in mice inoculated with EAC. The median lifespan and the number of animals that survived until the end of the experiment in each group are given in Table 1 and a graphical day-by-day representation of survival rate is shown in Figure 5. The presented data demonstrate that all animals from the control group died due to progressive tumor growth. The median lifespan of the control mice was 51.5 days and DMSO injection had no impact on both the lifespan and the survival rate in mice with EAC. The application of the non-selective cyclooxygenase inhibitor indomethacin or of the non-selective lipoxygenase pathway inhibitor NDGA did not increase either the survival rate or lifespan of mice with EAC. In each group receiving α-conotoxins MII or PnIA, only one animal out of eight (13%) survived; however, for mice that died during the experiment, the median lifespan increased by 20% and 81% in groups treated with α-conotoxins MII or PnIA, respectively. When baicalein was administered to mice, 50% of inoculated animals were alive after 200 days post inoculation; for dead animals, the median lifespan increased by 44% in this group.

The most pronounced antitumor effect was observed with the combined administration of α-conotoxin MII and indomethacin, as well as for the combination of α-conotoxin PnIA with baicalein. In the former group, four animals out of eight (i.e., 50%) were alive by the end of the experiment and, for dead mice, the median lifespan increased by 76%. In the latter group, the number of animals that survived by the end of the experiment was five out of eight (63%) and the median lifespan increased by 63%. 

## 3. Discussion

A large number of experimental studies support the hypothesis that activation of nAChRs inhibits apoptosis and promotes the survival of tumor cells. The most-studied tumor cells in this regard are those of lung cancer. Much is known about the biochemical pathways induced by nicotine through activation on nAChR and leading to increased tumor cell survival and other pro-oncogenic effects [6,10].

Similar data were obtained using tumor cells of other origins. In the MCF-7 breast cancer line, nAChR activation by nicotine led to a decrease in staurosporin-induced cell death, which was associated with activation of the α9 nAChR/STAT3/Galectin-3 pathway [11]. The reduced sensitivity of gastric cancer to cisplatin under the influence of nicotine was largely blocked by siRNA targeting the CHRNA5 gene, which encodes α5 subunit of nAChR [12]. Knockdown of α7 nAChR increased the sensitivity of gastric cancer cells to taxan and ixabepilone [13]. Nicotine contributed to the survival of pancreatic cancer cells, leading to protein kinase B (AKT) phosphorylation and activation of atypical protein kinase C, while mecamilamine, a non-specific nAChR blocker, reduced this effect [14].

In our experiments described above, nAChR blockers had a very weak cytotoxic effect or did not affect the viability of EAC cells in vitro. It can be concluded that in the absence of powerful apoptosis-inducing factors, a blockade of nAChRs does not lead to a noticeable increase in cell death.

It is well known that increased activity of cyclooxygenases, mainly of the inducible COX-2 enzyme, is associated with tumor growth. One of the consequences of increased COX activity is a decrease in the sensitivity of tumor cells to apoptotic signals. The amplification of apoptosis and the overall antitumor effect of both COX-1 and COX-2 inhibitors have been repeatedly described for a variety of transformed cells. For example, a non-selective COX inhibitor used in this study, indomethacin, suppressed the growth of human colon adenocarcinoma cells [15], MNNG/HOS osteosarcoma cells [16], human H4 and U87 glioma cells [17], and many other cell lines. The proapoptotic effect of COX inhibitors is associated with both reduced COX catalytic activity and inhibition of PGE2 synthesis, even with COX-independent mechanisms [18,19]. 

LOXs and the products of their catalytic activity also have an antiapoptotic effect and promote tumor growth. LOX inhibitors and, to a greater extent, drugs that inhibit the activity of both COX and LOX, have an antitumor effect [20,21]. Baicalein, NDGA and zileuton, the drugs of natural origin used in this work, possess the capacity to inhibit lipoxygenase pathways, which results in their antitumor activity. NDGA, a non-selective inhibitor of the LOX pathway, contributes to increased apoptosis in different cell lines including neuroblastoma [22], gastrointestinal cancer [2], as well as suppresses migration and metastasis of prostate cancer cells [23]. However, in some cases, the antitumor effect of NDGA was probably not associated with inhibition of lipoxygenases [22].

Baicalein, which specifically inhibits 12-LOX, induced apoptosis in pancreatic cancer cells [2]. In hepatocellular carcinoma, it induces apoptosis and autophagy, suppresses tumor cell proliferation in vitro, and inhibits the formation, growth, and metastasis in vivo [24]. A detailed analysis of the proapoptotic and antitumor effects of NDGA and baicalein on various types of tumor cells was undertaken by Yarla and coauthors [25].

Zileuton, a specific inhibitor of 5-LOX, manifested an antitumor effect against hepatocellular carcinoma in rats [26] and inhibited in vitro proliferation of colon cancer cells and tumor growth in a mouse [27]. In addition, it contributed to the suppression of systemic inflammation, reducing the number of polyps and their inflammatory infiltration in polyposis in mice [28].

In our experiments with EAC cells, COX inhibitors indomethacin, NS-398, and SC-560 induced only insignificant cell death or produced no effects. In contrast, LOX inhibitors NDGA and baicalein demonstrated a pronounced cytotoxic effect. This suggests that the survival of EAC cells is more dependent on the activity of the LOX pathway than on the COX pathway. The 5-LOX inhibitor zileuton had a weak cytotoxic effect against EAC cells, and this presumes that the cytotoxic effect of NDGA and baicalein results from the inhibition of 12-LOX, which is the target of these two drugs.

α-Conotoxins weakly influenced the cytotoxic effect of COX inhibitors (with one exception, which is discussed below) and significantly increased the effect of NDGA and baicalein. The data obtained suggested different roles of nAChR subtypes in enhancing the action of COX or LOX inhibitors. Thus, the cytotoxic effect of indomethacin was enhanced by conotoxin MII, a blocker of α6-containing and α3β2 nAChR subtypes. However, this conotoxin did not affect the action of NDGA and baicalein, while the conotoxins PnIA, RgIA, and ArIB11L16D, blockers of the α3β2, α9α10, and α7 nAChR subtypes, respectively, significantly increased the cytotoxic effect of these LOX inhibitors. This suggests that the role of α6-containing nAChR in the regulation of EAC survival may differ significantly from that of other non-neuronal nAChRs.

According to the available data [29,30,31], nAChRs upregulate COX-2 activity. Keeping this in mind, one can assume that the nAChR inhibitors decrease COX expression and therefore may enhance the action of COX inhibitors. At the same time, simultaneous application of nAChR blockers together with LOX inhibitors may lead to the suppression of both the COX and LOX pathways, thus resulting in a more significant suppression of the arachidonic acid cascade activity and in a more pronounced cytotoxic effect. 

In vivo, the applied drugs may exert effects not only on tumor cells, but also on the tumor microenvironment, affecting primarily antitumor immunity and neoangiogenesis. In mouse models, activation of nAChR has been shown to be associated with decreased immunity and increased susceptibility to chemical carcinogenesis in the lungs [32] and metastasis [33]. According to clinical studies, the risk of colorectal cancer is increased in smokers with reduced T-cell activity [34].

Increased COX and LOX levels are also associated with decreased antitumor immunity and increased angiogenesis [27,35]. Epidemiological data indicated that regular use of non-steroidal anti-inflammatory drugs, which are COX inhibitors, reduced the probability of developing cancer—in particular, colorectal cancer, head and neck cancer, as well as breast cancer. It is believed that the antitumor effect of non-steroidal anti-inflammatory drugs may be partly due to the inhibition of COX-2 and a decrease in the level of PGE2 in the tumor microenvironment [36].

Our results obtained in experiments on mice only partially match the results obtained in vitro. Thus, α-conotoxins PnIA and MII did show an antitumor effect in vivo; however, there was no significant antitumor effect of NDGA. This discrepancy may be explained by the action of these drugs on the cancer cell microenvironment as shown in our earlier work [9]. However, similar to the experiments in vitro, the combined use of α-conotoxin MII and indomethacin resulted in a more pronounced and prolonged inhibition of the EAC growth compared to the application of these drugs individually. In contrast, the combined application of α-conotoxin PnIA and indomethacin did not increase their antitumor effects, which can be explained by the different selectivity of the conotoxins MII and PnIA. We also revealed the antitumor effects of baicalein, which were increased by combined application of baicalein and α-conotoxin PnIA. Thus, we have found the synergistic effects of α-conotoxins and oxygenase inhibitors in the suppression of EAC growth.

## 4. Materials and Methods 

α-Conotoxins MII, PnIA, RgIA, and ArIB11L16D were obtained by solid-phase peptide synthesis according to [37]. α-Cobratoxin was isolated from Thailand cobra venom as in [38]. Indomethacin, SC-560, NS-398, nordihydroguaiaretic acid (NDGA), zileuton and baicalein were purchased from Merck KGaA (Darmstadt, Germany).

### 4.1. Viability of EAC Cells 

EAC cells were obtained sterile from the abdominal cavity of mice 8–10 days after intraperitoneal tumor inoculation. The concentration of cells in ascites fluid in vivo was 160–200 million/mLand the viability was 96%–99%. For the experiments, cell suspensions with a concentration of 0.25 million cells/mL (for analysis after 24 h) and 0.125 million cells/mL (for analysis after 48 h) were prepared using Dulbecco’s modified Eagle’s medium (Merck KGaA, Darmstadt, Germany) with the addition of 10% HyClone™ fetal bovine serum(Thermo Fisher Scientific, Waltham, MA, USA) and antibiotics (penicillin, streptomycin, and amphotericin B; all from Merck KGaA, Darmstadt, Germany). Preliminary studies have shown that EAC cells weakly proliferate in culture; after 48 h, most cells belong to the second and third generations (data not shown), but retain high viability for at least 48 h. In our experiments, cell viability in the control series averaged 91.8 ± 1.0% after 24 h (n = 55) and 82.3 ± 4.1% after 48 h (n = 45).

During the experiments, 200 mL of a cell suspension with a concentration of 0.25 million cells/mL were seeded into the wells of one of the 96-well culture plates, and 200 μL of a cell suspension with a concentration of 0.125 million cells/mL was seeded into the wells of the second plate.

nAChR blockers were dissolved in saline; COX and LOX inhibitors were dissolved in DMSO and then brought to the required volume with DMEM. nAChR blockers were added to the cell suspension at a volume of 25 μl, then to a final concentration of 1 nM, and COX or LOX inhibitors were added at a volume of 25 μl to the following final concentrations: indomethacin—10 μM, SC-560—0.1 μM, NS-398—5 μM, NDGA—30 μM, zileuton and baicalein—50 μM. In the control series, a similar amount of solvent was added. The final concentration of DMSO was 0.1% in all wells. In each series, n = 5, unless otherwise specified (in some cases, combined data from several experiments are presented, each with n = 5). The plates with cells were placed in a CO_2_ incubator at 37 °C and 5% CO_2_. After 24 and 48 h, the cells were collected in flow cytometry tubes from the first and second plates, respectively. Cell viability was evaluated by staining with propidium iodide (Sigma). Cell samples were analyzed using a flow cytometer BD FACSCanto II with Diva 7.0 software (Becton Dickinson, Franklin Lakes, NJ, USA).

### 4.2. EAC Growth

Experiments were carried out on Af/WySnMv mice, maintained at the vivarium of the Institute of Physiology of the National Academy of Sciences of Belarus. All studies were approved by the Commission on Bioethics of the Institute of Physiology of the National Academy of Sciences of Belarus. The ethical approval code is No. 1 of 31.01.2019.

Mice were inoculated subcutaneously with EAC by injecting 20 million viable cells in a volume of 0.2 ml. Starting from the day of inoculation, animals were injected intraperitoneally for 4 weeks with an interval of 3–4 days (twice a week) with the following final doses: nAChR blockers dissolved in isotonic sodium chloride at a dose of 1 nM/kg; indomethacin with a dose of 10 μM/kg, NDGA—30 μM/kg, baicalein—50 μM/kg. Indomethacin, NDGA, and baicalein were dissolved in DMSO, then diluted to the required concentration with saline; the final dose of DMSO when administered to mice was 1 ml/kg of weight. In one of the experimental series, mice received the DMSO only, without other drugs. nAChR blockers and arachidonic acid cascade enzyme inhibitors were not mixed; they were injected separately at intervals of 5–10 min In each series, eight animals were used. The changes in tumor volume and animal lifespan were registered. The volume of the tumor was determined by the formula V = (a*b*c*π)/6, where: a, b, c are three mutually perpendicular diameters of the tumor, 1/6 π = 0.52 is a constant value and V is the volume of the tumor (in cm^3^).

### 4.3. Statistical Analysis 

Statistical analysis was performed using standard statistical methods and the Statistica 7 software package (TIBCO Software Inc., Palo Alto, CA, USA). The significance of differences between the series experiments on the viability of cells in vitro are calculated according to the Mann-Whitney test, on the dynamics of tumor growth in vivo according to the Mann-Whitney test and the Nested ANOVA method, on mice survival according to the Cox’s F-test. Differences between the series were considered significant at *p* < 0.05.

## 5. Conclusions

The data obtained in this work indicate that α-conotoxins can enhance the cytotoxic effect of cyclooxygenase and lipoxygenase inhibitors against EAC cells. In vitro, α-conotoxin MII, blocking α3β2 and α6-containing nAChR subtypes, enhanced the cytotoxic effect of indomethacin, while α-conotoxins PnIA, RgIA, and ArIB11L16D, blockers α3β2/α6β2, α9α10, and α7 nAChR subtypes, respectively, as well as α-cobratoxin, a blocker of muscle and α7 nAChR subtypes, increased the cytotoxic effects of NDGA and baicalein. In vivo, the inhibition of EAC growth and the increase in mouse survival rate were promoted by baicalein, conotoxins MII and PnIA, but to an even greater extent by the combined use of conotoxin MII with indomethacin or conotoxin PnIA with baicalein. Thus, we showed, for the first time, an antitumor synergism of alpha-conotoxins and inhibitors of the arachidonic acid cascade.

## Figures and Tables

**Table 1:** The effects of α-conotoxins, COX and LOX inhibitors as well as their combinations on the lifespan and survival rate of animals carrying EAC.

Experimental Group	Number of Survived Mice (from Total 8 in the Test)	Lifespan of Animals after Tumor Inoculation, Days ^1^	
		Median (25−75)	Mean ± SEM
Control	0	51.5 (34.0 ÷ 56.8)	49.4 ± 6.0
DMSO	0	51.0 (33.0 ÷ 55.8)	48.9 ± 7.8
MII	1 ^2^	62.0 (54.0 ÷ 142.0)	92.9 ± 20.9
PnIA	1 ^2^	93.0 (53.0 ÷ 128.0)	93.0 ± 19.1
NDGA	0	43.5 (39.0 ÷ 67.3)	54.3 ± 9.0
Bai	4 ^2^	74.0 (52.0 ÷ 97.5)	75.5 ± 19.8
IM	0	48.5 (26.0 ÷ 88.8)	55.5 ± 11.6
IM + MII	4 ^2^	90.5 (54.0 ÷ 128.0)	91.3 ± 22.8
NDGA+MII	0	43.0 (35.0 ÷ 66.8)	53.3 ± 9.5
NDGA+PnIA	0	52.5 (40.0 ÷ 84.8)	60.3 ± 9.1
Bai+PnIA	5 ^2^	84.0 (58.0 ÷ 123.0)	92.3 ± 38.0
IM+PnIA	0	52.5 (22.0 ÷ 85.5)	56.9 ± 13.1

^1^ The lifespan was calculated for the animals that died during experiment (within 200 days); ^2^ *p* < 0.05 compared to control (according to the Cox’s F-test).

## Supplementary Materials

The following are available online at https://www.mdpi.com/1660-3397/18/4/193/s1, Figure S1: The effect of nAChR blockers on the viability of EAC cells after cultivation for 24 h, n = 55 (**a**) and 48 h, n = 45 (**b**). * *p* < 0.05 compared to control. Figure S2: The effect of NS-398 and nAChR blockers on the viability of EAC cells after cultivation for 24 h and 48 h (**b**). * *p* < 0.05 compared to control. Figure S3: The effect of SC-560 and nAChR blockers on the viability of EAC cells after cultivation for 24 h and 48 h (**b**). * *p* < 0.05 compared to control. Figure S4: The effect of zileuton (Zil) and nAChR blockers on the viability of EAC cells after cultivation for 24 h, n = 25 (**a**) and 48 h, n = 20 (**b**). * *p* < 0.05 compared to control; ** *p* < 0.05 compared to the Zil series.
