# Supplementary material for: α-Conotoxins Enhance both the In Vivo Suppression of Ehrlich carcinoma Growth and In Vitro Reduction in Cell Viability Elicited by Cyclooxygenase and Lipoxygenase Inhibitors
Source: Mar Drugs. 2020 Apr 7;18(4):193. doi: 10.3390/md18040193 (PMC7230841; doi:10.3390/md18040193)
Supplement: Supplementary file 1 [file marinedrugs-18-00193-s001.pdf]

Supplementary Materials

α-Conotoxins Enhance both the In Vivo Suppression of Ehrlich carcinoma Growth and In Vitro Reduction in Cell Viability Elicited by Cyclooxygenase and Lipoxygenase Inhibitors

Alexey V. Osipov [1], Tatiana I. Terpinskaya [2], Tatsiana Yanchanka [2], Tatjana Balashevich [2], Maxim N. Zhmak [1], Victor I. Tsetlin [1] and Yuri N. Utkin [1*]

[1] Shemyakin-Ovchinnikov Institute of Bioorganic Chemistry, Russian Academy of Sciences, ul. Miklukho-Maklaya 16/10, Moscow 117997, Russia; osipov-av@ya.ru (A.V.O.); mzhmak@gmail.com(M.N.Z.); victortsetlin3f@gmail.com (V.I.T.)

[2] Institute of Physiology, National Academy of Sciences of Belarus, ul. Akademicheskaya, 28, Minsk 220072, Belarus; terpinskayat@mail.ru (T.I.T); tanyaya190@gmail.com (T.Y.); tbalashevich@bk.ru (T.B.)

* Correspondence: utkin@mx.ibch.ru; yutkin@yandex.ru; Tel: +7 495 3366522

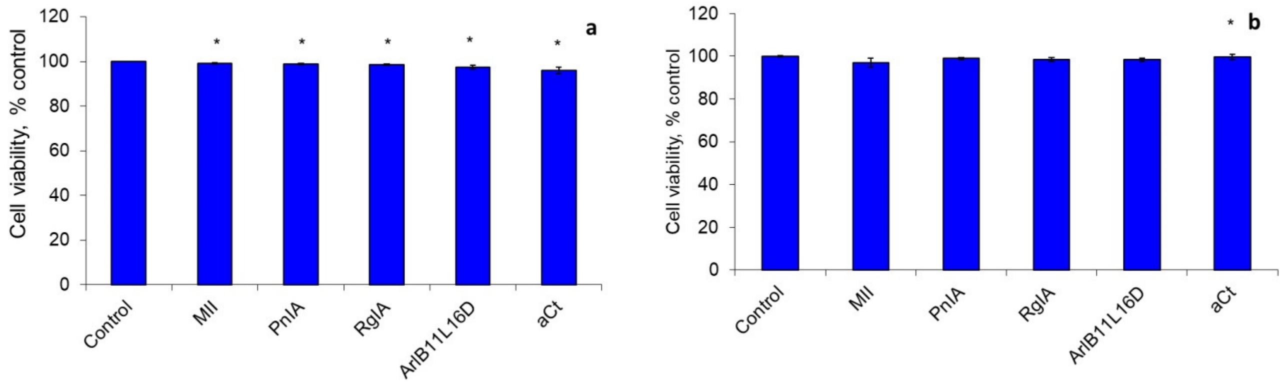

Figure 1. The effect of nAChR blockers on the viability of EAC cells in 24 h, n = 55 (a) and 48 h, n = 45 (b) in vitro; * P <0.05 when compared with control. aCt here and below is α-cobratoxin.

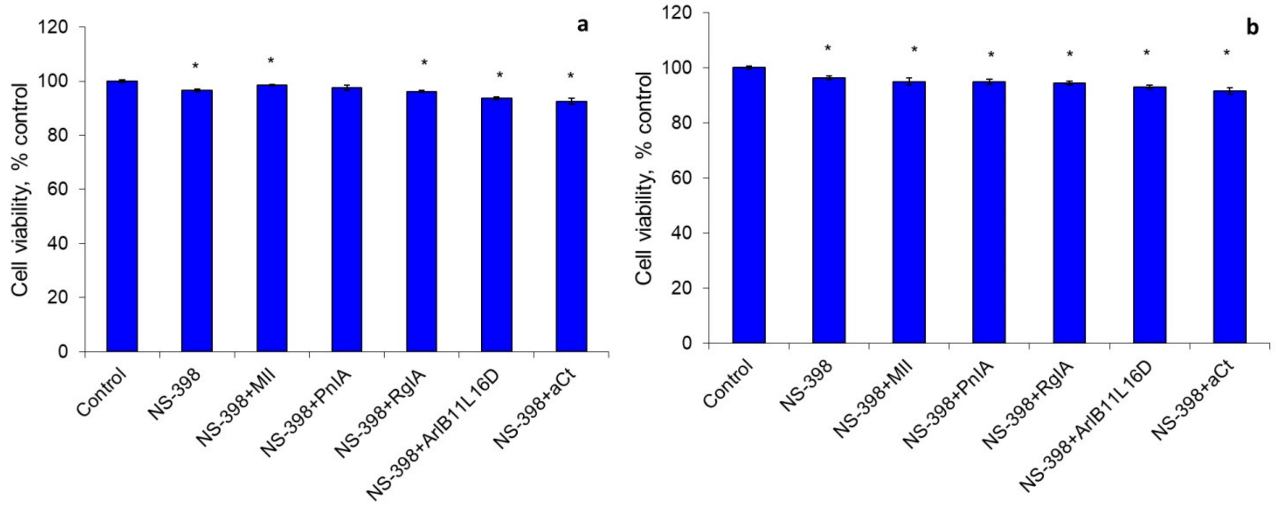

Figure 2. The effect of NS-398 and nAChR blockers on the viability of EAC cells in 24 h and 48 h (b) in vitro; * P <0.05 when compared with control.

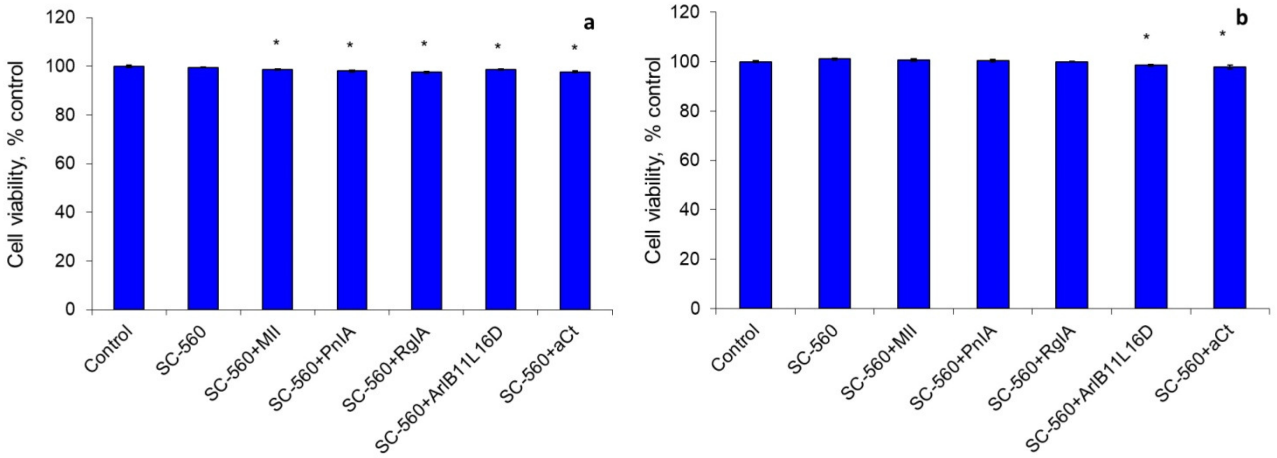

Figure 3. The effect of SC-560 and nAChR blockers on the viability of EAC cells in 24 h and 48 h (b) in vitro; * P <0.05 when compared with control.

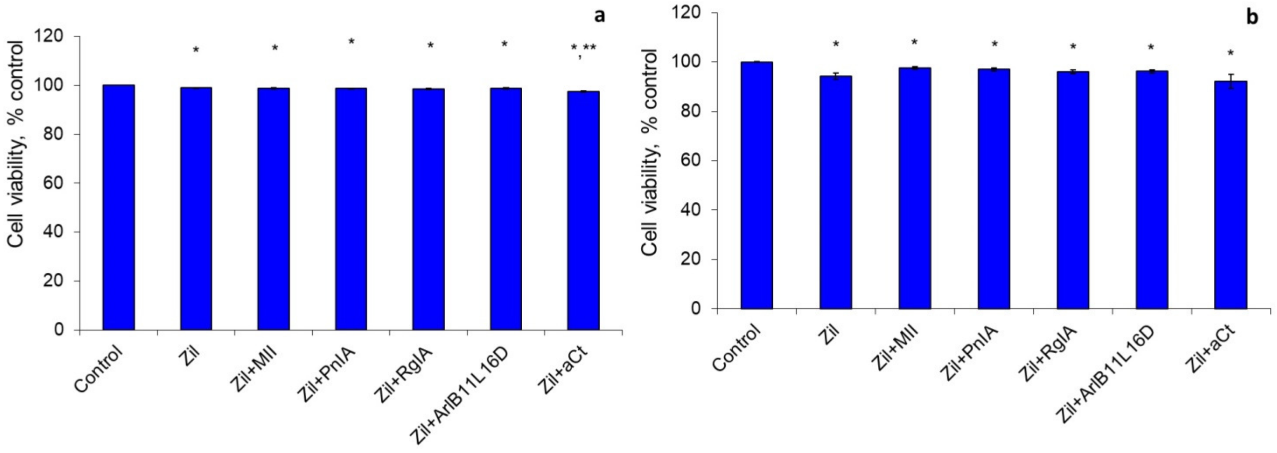

Figure 4. The effect of zileuton (Zil) and nAChR blockers on the viability of EAC cells in 24 h, n = 25 (a) and 48 h, n = 20 (b) in vitro; * P <0.05 when compared with control; ** P <0.05 when compared with the series " Zil ".
